# Supplementary material for: Protocol for an adaptive platform trial of intended service user-derived interventions to equitably reduce non-attendance in eye screening programmes in Botswana, India, Kenya and Nepal
Source: BMJ Open. 2025 Feb 2;15(1):e085353. doi: 10.1136/bmjopen-2024-085353 (PMC11792273; doi:10.1136/bmjopen-2024-085353)
Supplement: online supplemental file 2 [file bmjopen-15-1-s002.docx]

**Table S1. Expected error rates and sample size, by true effect difference between arms (d)**

| True effect difference between arms (d) | Type I error (α) | Type II error (β) | Median sample size [IQR] |
| --- | --- | --- | --- |
| 0% | 32.1% |  | 19,950 [3075,43525] |
| 1% |  | 8.4% | 8,150 [2500,22650] |
| 2% |  | 3.1% | 3,800 [1500,8100] |
| 3% |  | 1.8% | 2,100 [1000,4100] |
| 4% |  | 0.4% | 1,600 [900,2700] |
| 5% |  | 0.6% | 1,200 [700,2000] |
| 10% |  | 0% | 500 [400,800] |
| 15% |  | 0% | 300 [300,400] |
| 20% |  | 0% | 200 [200,300] |
| 25% |  | 0% | 200 [200,200] |
