# Supplementary material for: Protocol for an adaptive platform trial of intended service user-derived interventions to equitably reduce non-attendance in eye screening programmes in Botswana, India, Kenya and Nepal
Source: BMJ Open. 2025 Feb 2;15(1):e085353. doi: 10.1136/bmjopen-2024-085353 (PMC11792273; doi:10.1136/bmjopen-2024-085353)
Supplement: online supplemental file 3 [file bmjopen-15-1-s003.docx]

# Participant Information Leaflet

**Title: Protocol for an adaptive platform trial of intended service user-derived interventions to equitably reduce non-attendance in eye screening programmes in Botswana, India, Kenya & Nepal**

## Invitation

We would like to invite you to participate in this research study because you have been referred to a local treatment outreach centre. Joining the study is entirely up to you. This form explains the important things you should consider before deciding to join the study. Please ask the person conducting your eye screening any questions you still have before you decide whether to participate.

**Purpose of the research:** Only around half of the people found to have an eye problem and referred to local treatment clinics actually attend. The aim of this study is to see whether <*specify intervention*> improves attendance.

**Study location:** <*Specify study location*>

**What does my participation involve?** If you decide to take part then you will be randomly allocated to one of the two groups, as described below. There is no way to predict which group you will be allocated to. Like flipping a coin, there will be an equal chance of being placed in either group. Neither you nor the person screening and delivering this information to you can choose what group is chosen. You will be told which group you are to get.

*Group 1:* If you are randomized to Group 1 you will receive <*describe what would be delivered to the control group in each specific trial, considering intervention chosen*>

*Group 2:* If you are randomized to Group 2 you will receive <*specify intervention and describe what exactly would be delivered to participants in the intervention group*>

**Duration of participation:** <*specify duration of participation considering the intervention selected*>

**What data will we collect?** We will record whether or not you manage to attend the treatment clinic on your appointed date. We will use this data to assess whether attendance rates are different between the two groups.

**What are the risks of taking part?** Taking part in the study will have no impact on the care you receive., nor will you be exposed to any risks. Should you face any discomfort you are free to withdraw from the study any time by telling the screener or contacting the study leaders. Withdrawing will not affect the care you receive.

**What are the benefits?** There are no direct benefits or reimbursement for taking part. The information we learn from this study will hopefully help to improve the screening programme, increasing the chances that other people get the eye care they need in the future.

**Will my information be kept confidential?:** We will anonymise your data and keep it safe and secure on password-protected computers in <*specify country where trial is taking place*> and the UK. We will not sell your data. We will have your name removed so that you cannot be recognised and your data will have a code number instead. We will not share your name, date of birth, address, phone number or any other identifying information. When the study is completed, we will write-up our findings and publish them online in a medical journal so that other researchers can learn about what we found and use the information to help people in other places.

**Do I have to participate?:** You are being asked to volunteer to participate in this research study. It is up to you whether you choose to participate or not. There are no penalties and you will not lose anything if you decide not to join, or if you decide to withdraw from the study after you join. If you withdraw, we will not use your attendance data in our assessment.

## Who has reviewed this study?

This research study has been reviewed and approved by <*specify local board that provided approvals*>, and the London School of Hygiene & Tropical Medicine ethics committee. If you have any questions about your rights as a research participant, you may contact: <*provide contact details of local contact person for research queries*>

**Further information and contact details:** If you have any questions, you can ask anyone from our team now or later. If you have questions later, you may contact <*provide contact details for local research manager*>

**Consent form**

Name of participant

Date

| **Statement** | **Tick-box** |
| --- | --- |
| *I confirm that I have understood the information provided about the research study*  *assessing <specific to intervention> to increase attendance at eye treatment centres* |  |
| *I have had the opportunity to consider the information, ask questions and have these*  *questions answered satisfactorily* |  |
| *I understand that my anonymous data may be shared with other researchers or online in*  *a public repository, and that I will not be identifiable from this information* |  |
| *I understand that my participation is voluntary and that I am free to withdraw at any time*  *without having to give a reason* |  |
| *I understand that my decision will not affect the care that I receive* |  |

[ ] I consent

[ ] I do not consent

# Additions for obtaining consent with an impartial witness

*These additional elements are required for participants who cannot read*

Name of Impartial Witness: Date:

| **Statement** | **Tick-box** |
| --- | --- |
| *I confirm that the above-named participant has provided their free and fully informed*  *consent* |  |

***Signed statement from the person seeking consent***

Name of the person seeking consent

Date:

| **Statement** | **Tick-box** |
| --- | --- |
| *I confirm that I have explained the information provided about the research study accurately to the participant in their preferred language English. I confirm that, to the best of my knowledge, this information was understood by the participant and that she/he has freely given their consent to participate in the presence of*  *the above-named impartial witness* |  |
