# Supplementary figures and images for: Protocol for an adaptive platform trial of intended service user-derived interventions to equitably reduce non-attendance in eye screening programmes in Botswana, India, Kenya and Nepal
Source: BMJ Open. 2025 Feb 2;15(1):e085353. doi: 10.1136/bmjopen-2024-085353 (PMC11792273; doi:10.1136/bmjopen-2024-085353)

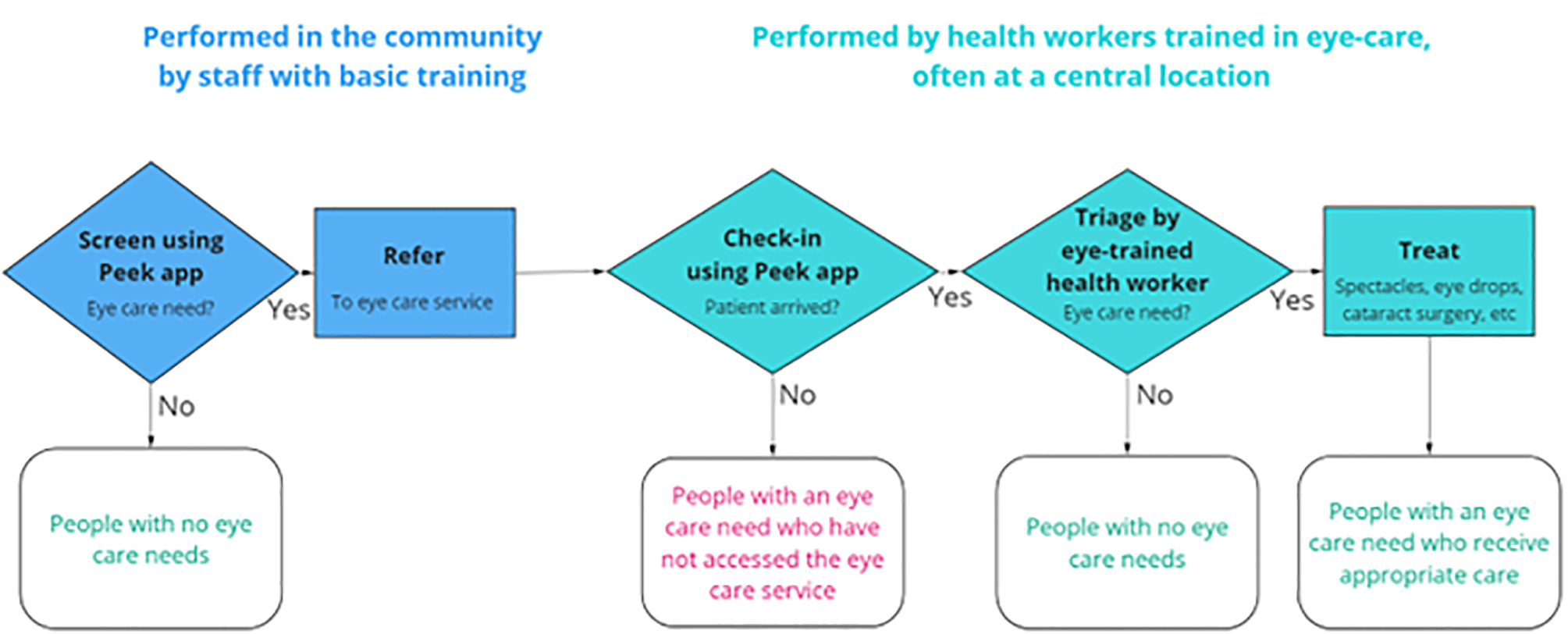

Supplement: online supplemental figure 1 [file bmjopen-15-1-s004.tif]

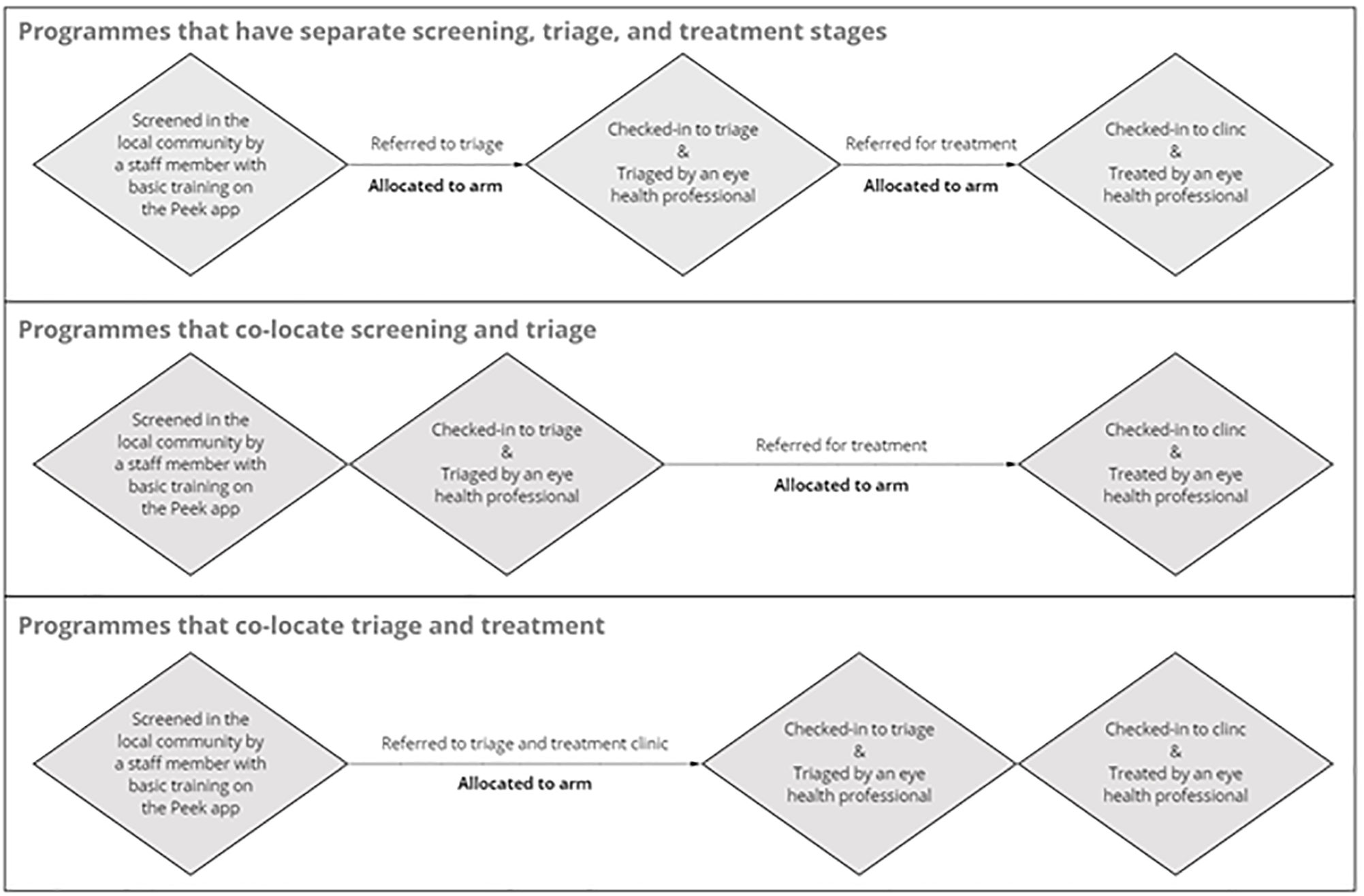

Supplement: online supplemental figure 2 [file bmjopen-15-1-s005.tif]

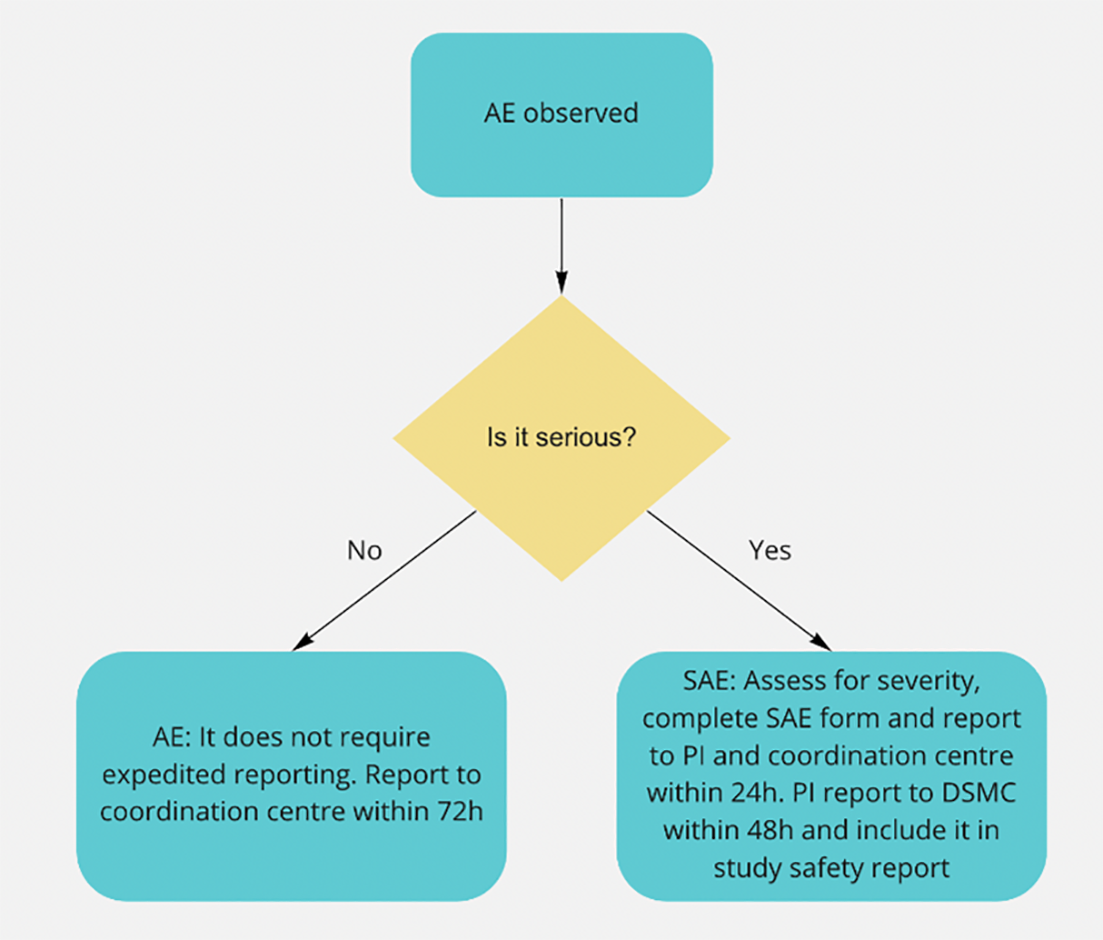

Supplement: online supplemental figure 3 [file bmjopen-15-1-s006.tif]
