# Supplementary material for: Protocol for an adaptive platform trial of intended service user-derived interventions to equitably reduce non-attendance in eye screening programmes in Botswana, India, Kenya and Nepal
Source: BMJ Open. 2025 Feb 2;15(1):e085353. doi: 10.1136/bmjopen-2024-085353 (PMC11792273; doi:10.1136/bmjopen-2024-085353)
Supplement: Supplementary file 7 [file bmjopen-2024-085353.reviewer_comments.pdf]

PEER REVIEW HISTORY

BMJ Open publishes all reviews undertaken for accepted manuscripts. Reviewers are asked to complete a checklist review form and are provided with free text boxes to elaborate on their assessment. These free text comments are reproduced below.

ARTICLE DETAILS

Title (Provisional)

Protocol for an adaptive platform trial of intended service user-derived interventions to equitably reduce non-attendance in eye screening programmes in Botswana, India, Kenya & Nepal

Authors

Allen, Luke; Kim, Min; Tlhajoane, Malebogo; Macleod, David; Nkomazana, Oathokwa; Gichangi, Michael; Mishra, Sailesh; Sabherwal, Shalinder; Carpenter, James R; Karanja , Sarah; Ho-Foster, Ari; Ratshaa, Bakgaki; Bolster, Nigel; Ramke, Jacqueline; Burton, Matthew J; Bastawrous, Andrew

---

VERSION 1 - REVIEW

**Reviewer**	**1**
**Name**	**Newman-Casey, Paula**
**Affiliation**	**University of Michigan, Ophthalmology & Visual Sciences**
**Date**	**22-Mar-2024**
**COI**	**None**

This is a very well written protocol description for a novel trial design in the public eye health space and I commend the authors for their innovative, important work with potential for really large impact on eye health outcomes.

**Reviewer**	**2**
**Name**	**Thykjær, Anne Suhr**
**Affiliation**	**Odense University Hospital, Ophthalmology**
**Date**	**28-May-2024**
**COI**	**None**

I thank BMJ Open for the opportunity to review this study protocol, that I found both well-written and promising.

I would have liked more details in Table 1, describing each screening programme; are they general screeningprogrammes, screening for ocular disease all round? Is the diseases screened for different in the included programmes?

Am I right in the understanding that you will include patients referred for follow up, and thus do not have data regarding never-attendees, meaning the people who are simply not referred in he first place? This might introduce a selection bias, where the most ressourceful are included in the study. Is it possible to collect information on the patiens who never attend? This would be valuable, however I recognize many pitfalls in getting to this data, and that it might no even be a possibility.

In "patient and public involvement" the aurthors mention advisory comittees, but fail to name the specific ones, maybe an elaboration would be possible?

Th authors describe adverse events and reporting of these. Which AE could the authors imagine occuring as no direct patientrelated interventions are done?

I am missing some references in the "limitations" section where a conceptual narrative of the Universal Health Coverage is mentioned, but no references are given.

VERSION 1 - AUTHOR RESPONSE

**Reviewer 1**		
9.	This is a very well written protocol description for a novel trial design in the public eye health space and I commend the authors for their innovative, important work with potential for really large impact on eye health outcomes.	No response required.
**Reviewer 2**		
10.	I would have liked more details in Table 1, describing each screening programme; are they general screening programmes, screening for ocular disease all round? Is the diseases screened for different in the included programmes?	The screening programmes in each country are run using the Peek smartphone app that screens for visual acuity and other common eye conditions such as red eye and cataracts. Therefore, screening is uniform in each setting with the exception being the study population targeted by the screening programme.<br><br>This is described on age 7 of the manuscript. We have added some further information. Table 1 has now been moved to page 7 (section on ‘Study setting’) to provide more information on how screening is conducted in each programme, before providing detail on the four study settings for the trial.

11.	Am I right in the understanding that you will include patients referred for follow up, and thus do not have data regarding never-attendees, meaning the people who are simply not referred in the first place? This might introduce a selection bias, where the most resourceful are included in the study. Is it possible to collect information on the patients who never attend? This would be valuable, however I recognize many pitfalls in getting to this data, and that it might no even be a possibility.	Yes, that is correct, participants for the trials will be those who have been screened and referred for onward care. Basic demographic data is collected for those who are screened and not referred, however, the data on sociodemographic characteristics that is used to determine the 'left behind' groups is only collected among those who are referred for onward care.<br><br>We have focused this project on the greatest drop-off in the treatment cascade, which is between screening and attending clinic. Internal data suggest that a relatively small proportion of the population in each setting are not screened. Future work could focus on this important group.
12.	In "patient and public involvement" the authors mention advisory committees, but fail to name the specific ones, maybe an elaboration would be possible?	We have added the specific committee names: 'The Pono Yame IM-SEEN advisory committee, the Kenya Vision Impact Programme IM-SEEN advisory committee, the NNJS IM-SEEN advisory committee, and the Dr Shroff's Charity Eye Hospital IM-SEEN advisory committee'
13.	The authors describe adverse events and reporting of these. Which AE could the authors imagine occurring as no direct patient related interventions are done?	Each of the trials would seek to test low-risk interventions that are unlikely to lead to any serious adverse events, and we cannot foresee any. However, inclusion of this section is mandated by our ethics committee and it's important that we have processes in place to handle any unforeseen issues.
14.	I am missing some references in the "limitations" section where a conceptual narrative of the Universal Health Coverage is mentioned, but no references are given.	We have added the following reference for this:<br><br>Allen LN. The philosophical foundations of 'health for all' and Universal Health Coverage. Int J Equity in Health. 2022.
